# A novel EGFR inhibitor, HNPMI, regulates apoptosis and oncogenesis by modulating BCL‐2/BAX and p53 in colon cancer

**DOI:** 10.1111/bph.16141

**Published:** 2023-09-12

**Authors:** Jeyalakshmi Kandhavelu, Kumar Subramanian, Vivash Naidoo, Giulia Sebastianelli, Phuong Doan, Saravanan Konda Mani, Hande Yapislar, Ebru Haciosmanoglu, Leman Arslan, Samed Ozer, Ramesh Thiyagarajan, Nuno R. Candeias, Clement Penny, Meenakshisundaram Kandhavelu, Akshaya Murugesan

**Affiliations:** ^1^ Division of Oncology, Faculty of Health Sciences University of the Witwatersrand Johannesburg South Africa; ^2^ Molecular Signalling Lab, Faculty of Medicine and Health Technology, BioMediTech Tampere University and Tays Cancer Centre Tampere Finland; ^3^ BioMediTech Institute and Faculty of Medicine and Health Technology Tampere University Tampere Finland; ^4^ Science Center Tampere University Hospital Tampere Finland; ^5^ Research and Publication Wing Bharath Institute of Higher Education and Research Chennai Tamil Nadu India; ^6^ Department of Physiology Acibadem University School of Medicine Atasehir, Istanbul Turkey; ^7^ Department of Biophysics Bezmialem Vakıf University School of Medicine Fatih, Istanbul Turkey; ^8^ Department of Physiology Bezmialem Vakıf University School of Medicine Fatih, Istanbul Turkey; ^9^ Department of Basic Medical Sciences, College of Medicine Prince Sattam Bin Abdulaziz University Al‐Kharj Kingdom of Saudi Arabia; ^10^ LAQV‐REQUIMTE, Department of Chemistry University of Aveiro Aveiro Portugal; ^11^ Faculty of Engineering and Natural Sciences Tampere University Tampere Finland; ^12^ Department of Biotechnology Lady Doak College Thallakulam, Madurai India

**Keywords:** alkylaminophenols, apoptosis, colon cancer, EGFR inhibitor, oncogenesis

## Abstract

**Background and Purpose:**

Colorectal cancer (CRC) is the second most lethal disease, with high mortality due to its heterogeneity and chemo‐resistance. Here, we have focused on the epidermal growth factor receptor (EGFR) as an effective therapeutic target in CRC and studied the effects of polyphenols known to modulate several key signalling mechanisms including EGFR signalling, associated with anti‐proliferative and anti‐metastatic properties.

**Experimental Approach:**

Using ligand‐ and structure‐based cheminformatics, we developed three potent, selective alkylaminophenols, 2‐[(3,4‐dihydroquinolin‐1(2*H*)‐yl)(*p*‐tolyl)methyl]phenol (THTMP), 2‐[(1,2,3,4‐tetrahydroquinolin‐1‐yl)(4‐methoxyphenyl)methyl]phenol (THMPP) and *N*‐[2‐hydroxy‐5‐nitrophenyl(4′‐methylphenyl)methyl]indoline (HNPMI). These alkylaminophenols were assessed for EGFR interaction, EGFR‐pathway modulation, cytotoxic and apoptosis induction, caspase activation and transcriptional and translational regulation. The lead compound HNPMI was evaluated in mice bearing xenografts of CRC cells.

**Key Results:**

Of the three alkylaminophenols tested, HNPMI exhibited the lowest IC_50_ in CRC cells and potential cytotoxic effects on other tumour cells. Modulation of EGFR pathway down‐regulated protein levels of osteopontin, survivin and cathepsin S, leading to apoptosis. Cell cycle analysis revealed that HNPMI induced G0/G1 phase arrest in CRC cells. HNPMI altered the mRNA for and protein levels of several apoptosis‐related proteins including caspase 3, BCL‐2 and p53. HNPMI down‐regulated the proteins crucial to oncogenesis in CRC cells. Assays in mice bearing CRC xenografts showed that HNPMI reduced the relative tumour volume.

**Conclusions and Implications:**

HNPMI is a promising EGFR inhibitor for clinical translation. HNPMI regulated apoptosis and oncogenesis by modulating BCL‐2/BAX and p53 in CRC cell lines, showing potential as a therapeutic agent in the treatment of CRC.

**Abbreviations**
CRC: colorectal cancer
HNPMI: *N*‐[2‐hydroxy‐5‐nitrophenyl(4′‐methylphenyl)methyl]indoline
IC_50_
: half‐maximal inhibitory concentration
THMPP: 2‐[(1,2,3,4‐tetrahydroquinolin‐1‐yl)(4‐methoxyphenyl)methyl]phenol
THTMP: 2‐[(3,4‐dihydroquinolin‐1(2*H*)‐yl)(*p*‐tolyl)methyl]phenol

**What is already known**

- Polyphenols modulate EGFR signalling pathways.
- Different staged CRC cells are resistant to known chemotherapeutic agents.

**What does this study add**

- The novel polyphenol derivative HNPMI might serve as an EGFR inhibitor in CRC cell lines.
- HNPMI reduced growth of CRC cell lines, in vitro and in vivo.

**What is the clinical significance**

- HNPMI may serve as a candidate compound for treating different stages of CRC.
- HNPMI may be considered as an example of EGFR‐targeted therapy for colorectal cancer.

## 1 INTRODUCTION

Colorectal cancer (CRC) is the second most lethal cancer and the third most malignant tumour worldwide (Xie et al., 2020). In recent years, there were significant advances in medical interventions including surgery, radiotherapy and systematic therapy for CRC. The negative prognostic factors are the stage of diagnosis, intolerance to surgery and unresectable lesions, leading to tumour‐related deaths (Keum & Giovannucci, 2019). Hence, an alternative therapy is required to improve and prolong the survival of the affected patients.

Targeted therapy can potentially inhibit the cell proliferation, differentiation and migration of cancerous cells. Indoline‐ and phenol‐containing natural products have been reported to confer antioxidant, pro‐oxidant, radical scavenging, antiviral, antibacterial, antimutagenic, anti‐inflammatory, anticarcinogenic and antitumour activities (Cai et al., 2004; Nandi et al., 2007; Selassie et al., 1999). Notably, eugenols (Pisano et al., 2007), hydroxycinnamic acids (caffeic and chlorogenic acids) (Gao et al., 2000; Rocha et al., 2012), curcumin (Chang et al., 2014; Jin et al., 2009) and honokiol (Battle et al., 2005) have been shown to exert anti‐tumour and anti‐proliferative effects on various cancers in preclinical models. A few of these compounds have been successful in clinical trials in patients.

Many nitrogen‐containing heterocyclic compounds form an integral part of pharmacologically active biomolecules and are known for their valuable therapeutic activity (Young, 1975). A range of heterocyclic compounds such as ibrutinib (Kaur et al., 2017), capecitabine (Kaur et al., 2017), folinic acid (Vendrusculo et al., 2018) and lapatinib (Alagarsamy et al., 2018) function as a potential anti‐cancer drugs against various types of cancer. Digalloylresveratrol, a synthetic ester of naturally occurring polyhydroxyphenols, inhibits the growth of human colorectal adenocarcinoma by interfering with the cell division from S phase to G2/M phase (Bernhaus et al., 2009). Polyphenols such as curcumin and silymarin have an anti‐proliferative effect on CRC by inducing apoptosis through the p53 pathway and the Wnt signalling pathway (Montgomery et al., 2016; Ramasamy & Agarwal, 2008).

We have previously synthesized a number of alkylaminophenols, of which 2‐[(3,4‐dihydroquinolin‐1(2*H*)‐yl)(*p*‐tolyl)methyl]phenol (THTMP), *N*‐[2‐hydroxy‐5‐nitrophenyl(4′‐methylphenyl)methyl]indoline (HNPMI) and 2‐[(1,2,3,4‐tetrahydroquinolin‐1‐yl)(4‐methoxyphenyl)methyl]phenol (THMPP) were identified as potential inhibitors of different types of cancer, including human osteosarcoma, glioblastoma and breast cancer (Doan et al., 2016a, 2020; Karjalainen et al., 2017; Palanivel et al., 2020). Each derivative exerted its anti‐cytotoxicity effect in different cancer cell lines, according to the sensitivity of each cell line. Further progress has been made in understanding how inhibition of the EGFR pathway and its signalling mechanism, by these compounds, leads to inducing apoptosis and cell cycle arrest, thus suppressing invasion and metastasis (Viswanathan et al., 2019). However, the role(s) of these EGFR inhibitors in regulating the genes and proteins involved in apoptosis and oncogenesis is not well studied in any of the cancer types, including CRC. In the present study, we aimed to understand the role(s) of the alkylaminophenols in modulating the EGFR signalling pathway in CRC cells. From studies with the three alkylaminophenols (THTMP, HNPMI, THMPP), we found that HNPMI regulated the EGFR pathway via the BCL‐2/BAX and p53 signalling cascades in CRC cells, thus inducing intrinsic apoptosis and affecting oncogenesis.

## 2 METHODS

### 2.1 Cytotoxicity assay on different cancer cell lines

PC3 (RRID:CVCL_0035), HepG2 (RRID:CVCL_0027) and Caco‐2 (RRID:CVCL_0025) were received as a gift from Prof. Gabriella Marucci, University of Camerino, Italy. Human prostate cancer cell line (PC3) and human liver cancer cell line (HepG2) were cultured in minimum essential medium (MEM) supplemented with 0.025 mg·ml^−1^ of amphotericin B, 0.1 mg·ml^−1^ of streptomycin, 100 U·ml^−1^ of penicillin and 10% FBS. The colorectal adenocarcinoma cell line (Caco‐2) was cultured in DMEM supplemented with 0.025 mg·ml^−1^ of amphotericin B, 0.1 mg·ml^−1^ of streptomycin, 100 U·ml^−1^ of penicillin, 0.1 mM of non‐essential amino acids solution and 10% FBS (Sigma‐Aldrich, St. Louis, MO, USA).

The PC3, Caco‐2 and HepG2 cell lines were seeded at an initial density of 1 × 10^5^ cells·ml^−1^ in 12‐well plates. Following overnight adherence, the cells were incubated with varying concentrations of HNPMI, THMPP and THTMP (100, 75, 50, 25 and 10 μM) for 24 h. Dimethyl sulfoxide (DMSO) (0.1%) served as a negative control. Cytotoxic effects on these cells, as inhibition of growth, was calculated after Trypan blue staining using Countess II FL Automated Cell Counter (ThermoFisher Scientific Inc., Waltham, MA, USA). Based on this preliminary cytotoxicity analysis, HNPMI was selected as a potential compound for further study, using the colon cancer cell lines, HT‐29 and DLD‐1.

The HT‐29 and DLD‐1 cell lines were seeded at an initial density of 2 × 10^5^ cells per well in 6‐well plates containing Dulbecco's modified Eagle's medium (DMEM)/F‐12 medium. After the cells had reached 80% confluency, they were incubated with different concentrations of HNPMI (100, 75, 50, 25 and 10 μM), for 24 h. In these experiments, 5‐fluorouracil (5′‐FU), an FDA‐approved chemotherapeutic drug, was used as a positive control, and 0.1% DMSO as a vehicle control. A sigmoidal dose–response curve was calculated to determine the half‐maximal inhibitory concentration (IC_50_) using MATLAB 2013a logistic function. All the experiments were conducted with n = 6 independent values. The percentage of inhibition was calculated using Equation (1).

(1) $$\text{Inhibition}\left(\%\right)=\frac{\text{Mean}\;\mathrm{No}.\text{of untreated cells(DMSO control)}-\text{Mean}\;\mathrm{No}.\text{of treated cells}}{\text{Mean}\;\mathrm{No}.\text{of untreated cells(DMSO control)}}\times 100$$

### 2.2 Molecular docking studies

To study the binding efficiency of the compounds THTMP, HNPMI and THMPP with the target EGFR, we performed molecular docking using PatchDock (RRID:SCR_017589) and SwissDock tools (RRID:SCR_022564) (Grosdidier et al., 2011; Schneidman‐Duhovny et al., 2005). The atomic coordinates of human EGFR human were obtained from the Protein Data Bank (PDB) (Berman et al., 2000). The obtained crystal structure was co‐crystallized with TGFα at a resolution of 2.5 Å by X‐ray crystallography (PDB ID: 1MOX). The structures of the three alkylaminophenol compounds were drawn in a molecular editor and converted into three‐dimensional coordinates. The molecular docking was performed by specifying the ligand‐binding site residues at Arg231 and Glu60 as observed in the crystal structure, which was performed after removing TGFα. Docking was performed using PatchDock, and further docked structures were compared based on the consensus region using SwissDock. The top 10 docked conformations were computed by PatchDock using docking score based on shape complementarity. The differences in CHARMM free energy of apo and ligand‐bound structures were analysed using SwissDock to predict the top docked conformations. The visual representation of the EGFR–HNPMI complex and its binding sites were plotted using Discovery Studio Visualiser 3.1 downloaded from www.accelerys.com (Accelerys, 2013).

### 2.3 Protein array analysis

The HNPMI‐treated cells were washed twice with phosphate‐buffered saline (PBS), and the protein was extracted using lysis buffer 17 provided in the Human Apoptosis Array Kit and Human XL Oncology Array Kit (R&D Systems, Minneapolis, MN, USA). The protein sample was extracted after gentle agitation at 4°C for 30 min and further centrifuged at 14,000× g for 5 min. The supernatant was collected, quantified using Qubit total protein assay (Thermo Fisher Scientific Inc., Waltham, MA, USA) and stored at −80°C for further analysis. The protein array for Human Apoptosis and Human XL Oncology, respectively, was performed according to the manufacturer's protocol. Briefly, the protein samples were added to the precoated nitrocellulose membrane. After overnight incubation at 4°C on a rocking shaker, the unbound proteins were washed twice with wash buffer. Respective arrays were incubated with a detection antibody cocktail for 1 h on a rocking platform shaker and washed thrice with wash buffer to remove the unbound antibodies. Membranes were incubated with Streptavidin‐HRP conjugate (R&D Systems) followed by chemiluminescence. Membranes were then digitally imaged with a chemiluminescent gel documentation and analysed using ImageJ Version 1.38e (National Institutes of Health, Bethesda, MD, USA). The pixel intensity of each spot (from n = 6 separate assays) was measured, and the data were normalized against reference spots by subtracting the average background signal from each spot. The fold change was calculated by comparing the treated samples with untreated control samples.

### 2.4 Cell cycle analysis using cell analyser

Colorectal adenocarcinoma cell line (HT‐29, RRID:CVCL_0320) was obtained from the ATCC Cell Biology Collection, Manassas, VA, USA, and the Dukes' type colorectal adenocarcinoma cell line (DLD‐1, RRID:CVCL_0248) was obtained from Health Science Research Resources Bank, Osaka, Japan, and was cultured in DMEM:nutrient mixture F‐12 (DMEM/F‐12) (Invitrogen, Waltham, MA, USA) supplemented with 10% v/v heat‐inactivated FBS (Invitrogen) and 100 μl of penicillin/streptomycin (100 IU·ml^−1^) (Lonza BioWhittaker®, Walkersville, MD, USA). All chemical components for the cell culture were purchased from Sigma‐Aldrich. The cells were maintained in a humidified culture incubator at 37°C supplemented with 5% CO_2_ and 95% O_2_. The entire study was performed with the cell passage number from 18 to 25 for both the CRC cell lines.

HT‐29 and DLD‐1 cells, at a density of 1 × 10^6^ cells·ml^−1^, were treated with 30 μM of HNPMI. The untreated group served as a control group. The cells were trypsinized, washed three times with PBS and resuspended in ~50 μl of PBS. The cells were then fixed with ice‐cold 70% ethanol and frozen at −20°C for 3 h prior to staining. Then the cells were washed and incubated with 200 μl of Muse™ Cell Cycle reagent (Merck Millipore, Burlington, MA, USA) for 30 min at room temperature in the dark. Samples were transferred into microcentrifuge tubes (1.5 ml) and analysed using the Muse™ Cell Analyser.

### 2.5 Apoptosis assay

Apoptosis assessment was conducted using Muse® Annexin V and Dead Cell Kit (Luminex Corporation, Austin, TX, USA). In brief, 2 × 10^5^ cells·ml^−1^ of HT‐29 and DLD‐1 cell lines were cultured in 6‐well plates with DMEM/F‐12 medium supplemented with 10% fetal bovine serum (FBS) and 1000 U/2 L of penicillin and streptomycin, until the cells reached 60%–70% confluence. Then the cells were starved overnight in medium without serum. After incubating the cells with HNMPI (30 μM) for 24 h, cells were trypsinized and pelleted. The cell pellets were resuspended in 100 μl of 1% bovine serum albumin (BSA) and 100 μl of the Muse® Annexin V and Dead Cell Reagent. The complete mix was further incubated for 20 min in the dark at room temperature. Finally, the cells were analysed using the Muse™ Cell Analyser to determine the percentage of cell death (Merck Millipore).

### 2.6 Real‐time RT‐PCR of apoptotic genes

RNA was extracted from 30 μM of HNPMI‐treated HT‐29 and DLD‐1 cells using the Direct‐zol™ RNA extraction kit (Zymo Research, Irvine, CA, USA) according to the manufacturer's instructions. cDNA was synthesized with 2 μg of RNA by reverse transcription reaction using the Applied Biosystems™ High‐Capacity RNA‐to‐cDNA kit (Catalogue Number 4387406). The thermal cycling condition for reverse transcription was optimized with the initial activation step for 2 min at 95°C, extension step of 37°C for 60 min, followed by a denaturation step at 95°C for 5 min. The real‐time PCR was performed (Applied Biosystems Informatics [ABI] 7500 Real‐Time PCR, Waltham, MA, USA) using QuantiTect SYBR Green one‐step qRT‐PCR kit (Qiagen N.V., Hilden, Germany) following the manufacturer's instructions. Glyceraldehyde phosphate dehydrogenase (GAPDH) was used as an endogenous reference. The following primer combination was used for the experiment: GAPDH, sense (5′‐TGCMTCCTGCACCACCAACT‐3′) and antisense (3′‐YGCCTGCTTCACCACCTTC‐5′); BAD, sense (5′‐ATCTCAGCTCCTCTAAGCCCC‐3′) and antisense (3′‐CCCTGAGCTTCCCCTCGATT‐5′); BAX, sense (5′‐TTCATCTCAGTCCCCTGCCC‐3′) and antisense (3′‐GGAGACAGGGACATCAGTCG‐5′); BCL‐2, sense (5′‐CCTGTGGATGACTGAGTACC‐3′) and antisense (3′‐GAGACAGCCAGGAGAAATCA‐5′); CASP3, sense (5′‐GCTCGCTAACTCCTCACGG‐3′) and antisense (3′‐TCCAATTCCTTTTCGGCCCTG‐5′); and p53, sense (5′‐GCTCGACGCTAGGATCTGAC‐3′) and antisense (3′‐CCCAGGGTTGGAAGTGTCTC‐5′). The reaction was completed with 40 cycles of annealing/extension step for 10 s at 60°C. The threshold cycle values (Ct) were normalized to the endogenous reference gene, GAPDH, and the relative fold change was calculated by the ΔΔCt method (Livak & Schmittgen, 2001).

### 2.7 Immunofluorescence and confocal imaging

Subcellular protein expression in control and drug‐treated cells was assessed using indirect immunofluorescence and imaging with confocal microscopy. Cells (1 × 10^3^ cells·ml^−1^) were grown on coverslips in petri dishes until reaching 70% confluence, then fixed in 30% v/v of formaldehyde (Sigma‐Aldrich) with PBS and permeabilized using 0.25% v/v of Triton X‐100 (Sigma‐Aldrich) in BSA/PBS (0.5% v/v). The cells were placed on microscope slides and incubated with primary antibody (1:200) in 0.5% v/v BSA/PBS, overnight at 4°C. Two different primary antibodies were used, anti‐BCL‐2 (C‐2) mouse monoclonal IgG (Santa Cruz Biotechnology, Inc, Dallas, TX, USA, Cat# sc‐7382, RRID:AB_626736) and anti‐p53 rabbit polyclonal IgG (R&D Systems, Cat# AF1355, RRID:AB_354749). After 1 h of incubation in the dark, the slide was incubated with Alexa Fluor® 488 conjugated donkey anti‐mouse (Molecular Probes, Winsford, UK, Cat# A‐21202, RRID:AB_141607) and Alexa Fluor® 488 conjugated donkey anti‐goat (Molecular Probes, Cat# A‐11055, RRID:AB_2534102) secondary antibodies (1:200 in 0.5% v/v BSA/PBS). The coverslip was stained further with 300 nM solution of 4′,6‐diamidino‐2‐phenylindole (DAPI) (1:10,000, Boehringer Mannheim, Mannheim, Germany) to localize the nuclei. The coverslip was then mounted on Gel Mount™ aqueous mounting medium (Sigma‐Aldrich). Digital images were obtained with a Zeiss LSM 780 laser scanning microscope using the 63× objective. Fluorescence staining was assessed qualitatively using the following scale: + +, minimal/low‐intensity fluorescence; ++ ++, medium‐intensity fluorescence; and +++ +++, high‐intensity fluorescence. The immuno‐related procedures used comply with the recommendations made by the *British Journal of Pharmacology* (Alexander et al., 2018).

### 2.8 Xenograft animal models and grouping

All animal care and experimental procedures complied with the EU guidelines and were approved by the Acibadem Mehmet Ali Aydinlar University Animal Experiments' Local Ethics Committee (No. 07/05/2018 ACU/HADYEK 2018/22). Animal studies are reported in compliance with the ARRIVE guidelines (Percie du Sert et al., 2020) and with the recommendations made by the British Journal of Pharmacology (Lilley et al., 2020).

HCT‐116 CRC cells (RRID:CVCL_0291; kindly provided by Dr B. Vogelstein, Johns Hopkins Kimmel Cancer Center, Baltimore, MD, USA) were used for the development of the xenograft animal model. In order to stably inherit the biological characteristics of the tumour tissue (Wang et al., 2017), 4‐ to 5‐week‐old nod‐scid male mice were selected for the study and purchased from the Acibadem Mehmet Ali Aydinlar University Laboratory Animal Research Center (DEHAM), Istanbul, Turkey. Their bait pellets, water, water bottle and cage were maintained under specific pathogen‐free conditions. All animals were housed in an environment in a 12/12‐h light/dark cycle with a temperature of 22 ± 1°C under a relative humidity of 45%–65%. HCT‐116 cells (1 × 10^6^ cells) in the logarithmic growth phase, were collected from the culture. Collected cells were resuspended in 1 ml of medium and inoculated subcutaneously into the right flank of the nude mice at a volume of 200 μl per mouse. The physiological conditions of the mice, and their diet and defecation were monitored on a daily basis. After the tumour volume had reached 70–100 mm^3^, animals were randomly divided into four groups (n = 8 per group): cancer negative control (CNC) group, cancer treatment group 1 (CTG1), cancer treatment group 2 (CTG2) and cancer treatment group 3 (CTG3).

### 2.9 In vivo testing of the lead compound

The CNC group was injected i.p. with 0.2 ml of normal saline, twice a week for a period of 4 weeks. The lethal dose and LD_50_ of HNPMI were calculated as 170 mg kg^−1^ and 150 mg kg^−1^, respectively. The mice in the CTG groups were injected i.p. with different doses of HNPMI (12 mg kg^−1^, 120 mg kg^−1^, 150 mg kg^−1^, in a volume of 0.2 ml), twice a week for the same period. HNPMI was dissolved in DMSO initially and further diluted with serum (1:9). The highest dose at which there was no deaths over 4 weeks was 120 mg kg^−1^ and is referred to as the safety dose; this dose was selected to study the effect of HNPMI against colon cancer xenografts in mice. During the treatment period, the weight of the mice and diameter of the tumour were measured twice a week to determine the signs of toxicity and effects of treatment. A standard vernier calliper was used for the measurement of tumour volume. Animals were killed by decapitation, 28 days from the day of the first injection. The tumour volume was calculated using Equation (2).

(2) $$\text{Vtumour}=\text{The formula}\;½\;\left(a\times \text{b2}\right)$$

where a and b represent the longest and shortest diameter of the tumour, respectively.

### 2.10 Data and statistical analysis

The data and statistical analysis comply with the recommendations of the *British Journal of Pharmacology* on experimental design and analysis in pharmacology (Curtis et al., 2022). For all in vitro analysis, statistical analysis was performed only for studies where each group size was n ≥ 5 independent values. In vivo studies were designed to generate groups of equal size (n = 8). Statistical analysis was performed only for studies where each group size was at least n ≥ 5 independent values. The variation in group size within an experiment was due to unexpected death of animals. GraphPad Prism Version 6.0 (RRID:SCR_002798) was used for the statistical analysis. Multiple comparisons of the significant ANOVA were assessed by two‐way ANOVA using Sidak's multiple‐comparisons test and Tukey's multiple‐comparisons test. Significant differences between the groups were analysed using Student's unpaired *t* test. Data are expressed as the mean ± SEM or mean ± SD, and *P* values < 0.05 were considered statistically significant.

### 2.11 Materials

**FIGURE 1:**
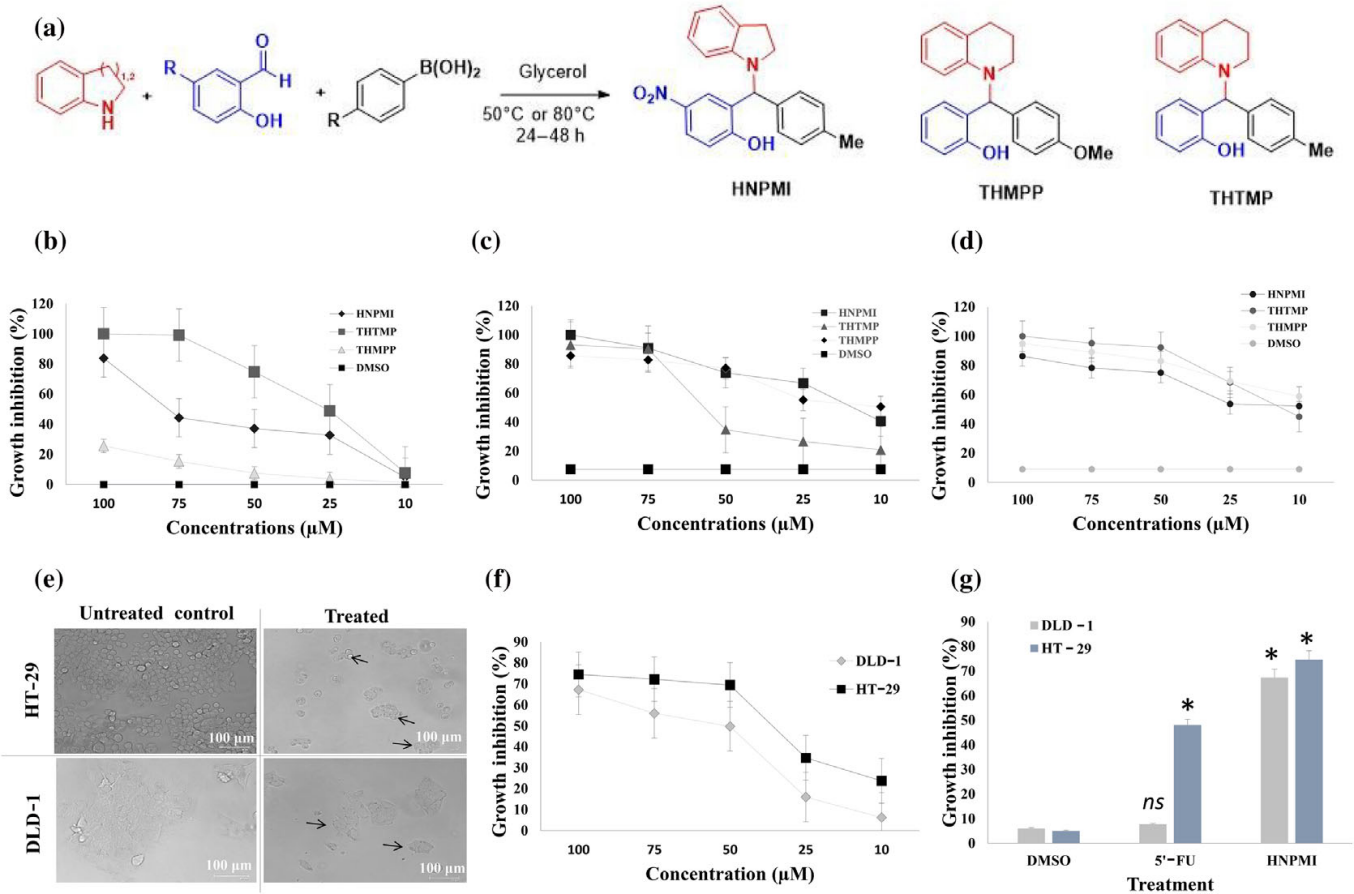
Synthesis of alkylaminophenols and their cytotoxic effects on different cancer cell lines (PC‐3, Caco‐2 and HepG2). (a) Synthesis of three alkylaminophenols, HNPMI, THTMP and THMPP. The effect of the three alkylaminophenols on the inhibition of growth of prostate cancer (PC3) cells (b), colon cancer cells (Caco‐2) (c) and lung cancer cells (d) HepG2, over a range of concentrations (10, 25, 50, 75 and 100 μM). (e) Microscopic images showing morphological disturbance of adenocarcinoma colon cancer cell lines (HT‐29 and DLD‐1) treated with the selected lead compound, HNPMI. (f) The growth inhibition in differently staged colorectal cancer (CRC) cells treated with HNPMI. Data of (b)–(d) and (f) represent mean ± SEM (n = 6 independent values). (g) Effects of HNPMI on the growth inhibition of CRC cells compared with that of the positive control 5‐fluorouracil (5′‐FU) at 100 μM and the negative control dimethyl sulfoxide (DMSO). Data shown are means ± SD, from n ≥ 5 experiments. *P ≤ 0.05, significantly different from DMSO treatment; ns, no significant difference between DMSO and treated samples.

The synthesis and spectral characterization of the three alkylaminophenols, HNPMI, THMPP and THTMP (Figure 1a), were carried out as described previously (Neto et al., 2016; Rosholm et al., 2015). The three phenolic compounds and the positive control 5′‐FU (Sigma‐Aldrich, St. Louis, MO, USA) were dissolved in DMSO (Sigma‐Aldrich) to obtain a stock solution of 100 mM. An intermediate dilution necessary for the respective experiments was prepared using stock solution. Details of the other materials and their suppliers are provided in specific subsections of the methods.

### 2.12 Nomenclature of targets and ligands

Key protein targets and ligands in this article are hyperlinked to corresponding entries in the IUPHAR/BPS Guide to PHARMACOLOGY (http://www.guidetopharmacology.org) and are permanently archived in the Concise Guide to PHARMACOLOGY 2021/22 (Alexander, Fabbro et al., 2021a,b; Alexander, Kelly et al., 2021).

## 3 RESULTS

### 3.1 Alkylaminophenols inhibited the cell growth of human prostate (PC‐3), colon (Caco‐2) and liver (HepG2) cancer cells

The cytotoxic effect of the alkylaminophenols, HNPMI, THTMP and THMPP (Figure 1a), was evaluated against cell lines from three different types of cancer, PC‐3, Caco‐2 and HepG2, over a range of concentrations (10, 25, 50, 75 and 100 μM). All compounds caused concentration‐dependent inhibition of cell proliferation towards these cell lines. At 100 and 75 μM, THTMP inhibited the growth of PC‐3 cells to about 100% (Figure 1b). HNPMI showed 84% of PC‐3 cell growth inhibition at 100 μM but with only 44.3% activity for THMPP. Figure 1c shows the inhibition of growth of Caco‐2 cells, which was directly proportional to the concentration of the compounds, more especially for HNPMI and THMPP than for THTMP. At 100 and 75 μM, HNPMI, THTMP and THMPP showed increased inhibition of cell growth to about 100%, 93.2% and 85.7%, respectively. As the concentration was lowered from 50 to 10 μM, THTMP inhibited Caco‐22 cells from <40% to 20%. Further analysis of the effect of these compounds on HepG2 cells showed a similar trend of results as observed for the other two cell lines (Figure 1d). Approximately, 90% of HepG2 growth inhibition was observed at 100 μM with a steady rate of decline to about 40% as the concentration was decreased to 10 μM.

The concentration–response curve was analysed to identify the IC_50_ value of HNPMI, THTMP and THMPP on PC‐3, Caco‐2 and HepG2 cancer cell lines (Table S1). Interestingly, HNPMI exhibited the lowest IC_50_ value of 28 ± 1.8 μM, specifically on the colon cancer cell line, Caco‐2. The response of CRC to treatment in patients is related to the stage of the disease, we therefore decided to validate the effect of HNPMI on cell lines derived from different stages of colon cancer. Although Caco‐2 was used for our preliminary analysis, it is not classified with a specific tumour stage. We chose the HT‐29 and DLD‐1 cell lines for further study, as DLD‐1 cells represent a late‐stage metastatic tumour derived from the large intestine of human colorectal adenocarcinoma, whereas HT‐29 cells derives from a mid‐stage human colon adenocarcinoma.

### 3.2 HNPMI induced morphological changes in adenocarcinoma colon cancer cell lines

HNPMI treatment induced morphological changes in both HT‐29 and DLD‐1 cells after 24 h of treatment. The untreated cells had intact cell membranes with appropriate nuclear morphology, suggesting normal proliferation. In comparison, the HNPMI‐treated HT‐29 and DLD‐1 cells showed morphological changes, which included the loss of cell adhesion, membrane shrinkage and membrane blebbing (Figure 1e). With increasing concentrations of HNPMI, growth inhibition also increased in both cell lines, indicating the concentration‐dependent inhibitory property of HNPMI (Figure 1f). From these experiments, the IC_50_ value of HNPMI was found to be 39.3 ± 7.03 μM for DLD‐1 cells and 31.9 ± 1.25 μM for HT‐29 cells, implying a need for a higher concentration of HNPMI for the late‐stage cancer cells, DLD‐1 (Kishida et al., 1998), than in the mid‐stage HT‐29 cells. At 100 μM concentration, HNPMI exhibited 65% and 70% of growth inhibition in DLD‐1 and HT‐29 cells, whereas exposure to the positive control compound 5′‐FU, showed only 10% and 50% of inhibition in DLD‐1 and HT‐29 cells, respectively (Figure 1g). Such differing effects might be due to the differently staged CRC cells and differing sensitivity to drug treatment (Inaba et al., 1998; Van der Jeught et al., 2018).

### 3.3 HNPMI–EGFR interaction modulated genes involved in tumour progression

**FIGURE 2:**
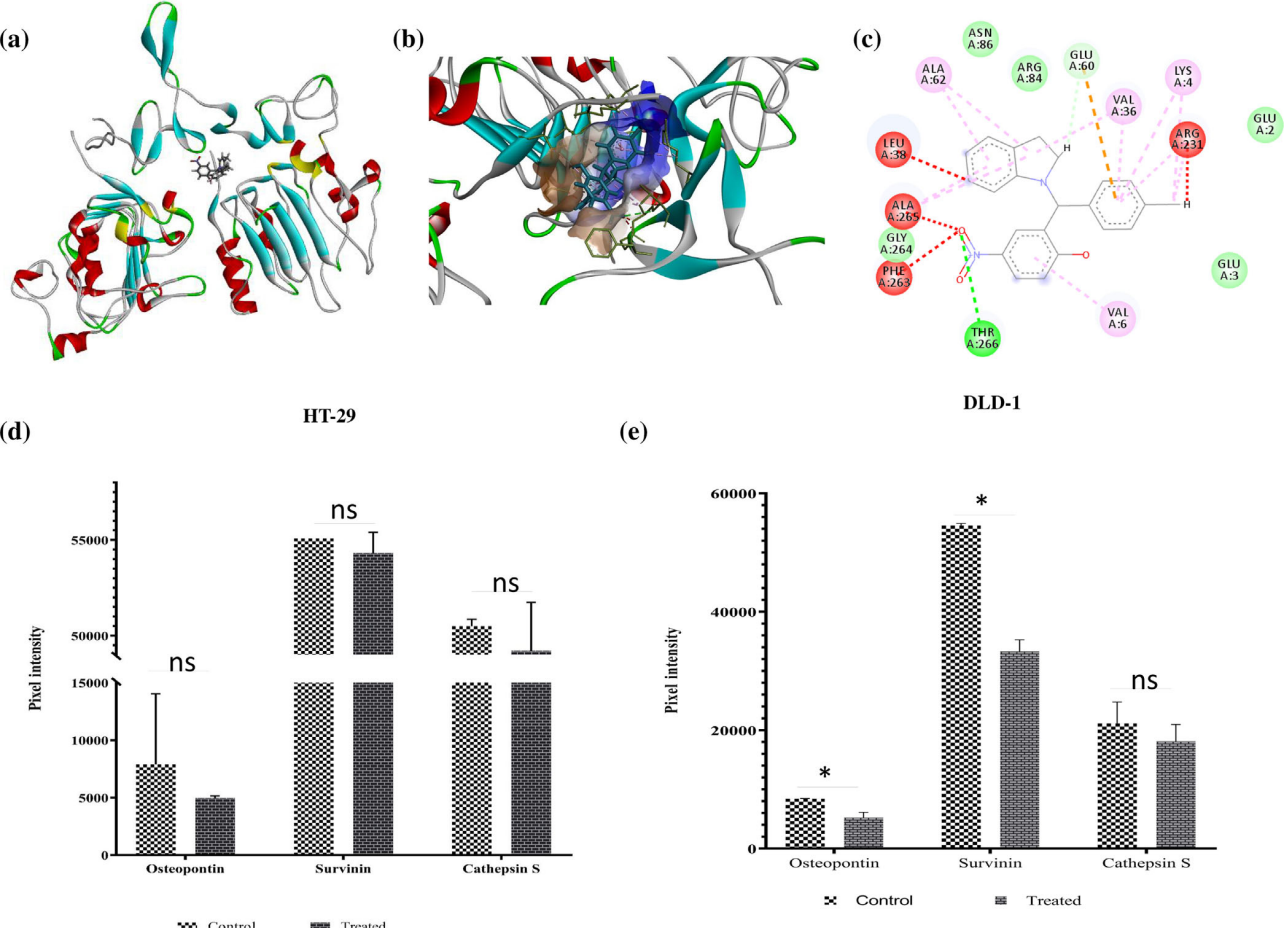
Docking of HNPMI–EGFR complex showed regulation of genes involved in tumour progression. (a) EGFR–HNPMI complex obtained from PatchDock docking program. (b) The zoomed view of the protein–ligand complex and the binding site is shown as the hydrophobic surface. (c) Two‐dimensional chemical interaction of EGFR–HNPMI. Human XL Oncology Array‐based protein expression assay representing the modulation of genes including osteopontin, survivin and cathepsin S, upon HNPMI–EGFR binding in (d) HNPMI‐treated HT‐29 cells and (e) DLD‐1 cells. Results shown are arbitrary units of intensity and are expressed as means ± SEM (n = 6). *P< 0.05, significantly different as indicated; ns, not significantly different, as indicated; Student's *t* test.

EGFR is the key regulator of various cellular events in many cancers, including in CRC. Our previous study has reported on the therapeutic role of HNPMI targeting EGFR and thus inferred it as a potential anti‐cancer agent against breast cancer (Palanivel et al., 2021). In our present analysis, we docked the atomic crystal structure of EGFR (PDB ID: 1MOX) to our lead indoline derivative, HNPMI. The mode of interaction, the affinity of HNPMI with EGFR and its influence on the downstream signalling pathway were studied. Protein–ligand complex with the patch dock score of 4538 was identified and showed stable interactions. This complex was validated further using SwissDock by calculating the free energy perturbation of molecules both in apo and in EGFR–HNPMI complex and was found to be −7.10 kcal·mol^−1^. Visual representation of the EGFR–HNPMI complex is shown in Figure 2a, and the ligand positioning the binding site of EGFR along with 2‐D ligand interaction and chemical interactions are illustrated in Figure 2b,c. The EGFR–HNPMI complex showed various types of interactions including Van der Waals, H‐bond and hydrophobic interactions. The complex displayed 10 Van der Waals hydrophobic interactions with the amino acids Phe263, Thr266 and Val36; two charged amino acid residue interactions with Arg231 and Glu60; and 30 H‐bond interactions. We presume that the presence of these many interactions may favour the binding of the ligand (HNPMI) to EGFR, leading to stronger interaction.

We further endeavoured to investigate the modulation of downstream signalling pathways regulating cell migration, invasion and apoptosis, following HNPMI–EGFR binding. To identify the differentially expressed proteins of EGFR pathway from HNPMI‐treated HT‐29 and DLD‐1 cells, the Human XL Oncology Array‐based protein expression assay was performed. Down‐regulation of osteopontin, survivin and cathepsin S was observed in DLD‐1 cells, validating the regulation of the EGFR pathway in these HNPMI‐treated cells (Figure 2d,e). Several reports have indicated that the up‐regulation of osteopontin (Wai & Kuo, 2008; Weber et al., 2010), survivin, an apoptosis inhibitor (Dong et al., 2015), and cathepsin S (Zhang et al., 2015) is associated with tumour cell migration, invasion, progression and metastasis. Thus, our data reflected the significant association of HNPMI and EGFR in down‐regulating these genes involved in CRC progression.

### 3.4 HNPMI induced G0/G1 arrest in HT‐29 and DLD‐1 cells

**FIGURE 3:**
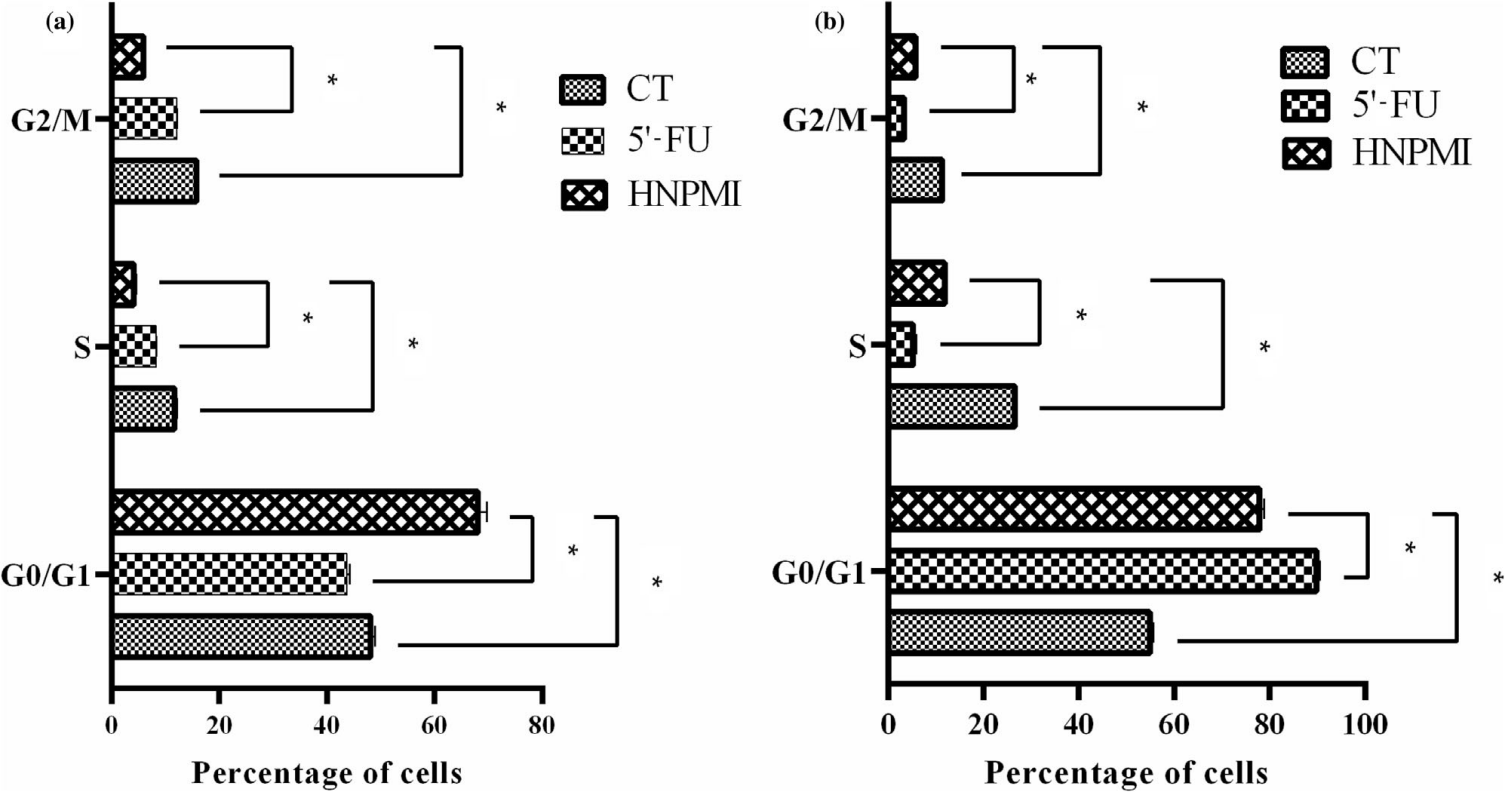
Cell cycle analysis of colorectal cancer (CRC) cells. The bar plot showing the percentage of the cells in G0/G1, S and G2/M phases of cell cycle before and after the treatment of (a) HT‐29 cells and (b) DLD‐1 cells with HNMPI (30 μM) for 24 h. 5‐Fluorouracil (5'‐FU; 100 μM) was used as a positive control and compared with untreated CRC cells that served as a negative control (CT). The data were calculated as the percentage of cells in each phase of the cell cycle and are shown as means ± SEM from n = 6 independent values. *P < 0.05, significantly different as indicated; two‐way ANOVA with multiple comparisons.

The anti‐proliferative activity of HNPMI on the cell cycle populations of HT‐29 and DLD‐1 cells was analysed using the Muse™ Cell Analyser. Cell analysis revealed changes in the distribution of cells in G0/G1 and, in turn, in the S and G2/M phases. With treatment, the cell population in G0/G1 phase increased significantly, indicating arrest at this time. The percentage of cells in G0/G1 increased in HNPMI‐treated HT‐29 (Figure 3a) and DLD‐1 cells (Figure 3b), compared with data from control cultures. This was accompanied by a significant decrease in cell in both S phase and G2/M phase to <11%. We have observed that 5'‐FU induced cell cycle arrest at G0/G1 phase at 100 μM in HT‐29 cells, in agreement with the previous reports (Grivicich et al., 2007; Jiang et al., 2017). These results showed that the cell cycle was arrested in the G0/G1 phase, following HNPMI–EGFR association.

### 3.5 HNPMI induced apoptosis in HT‐29 and DLD‐1 cells

**FIGURE 4:**
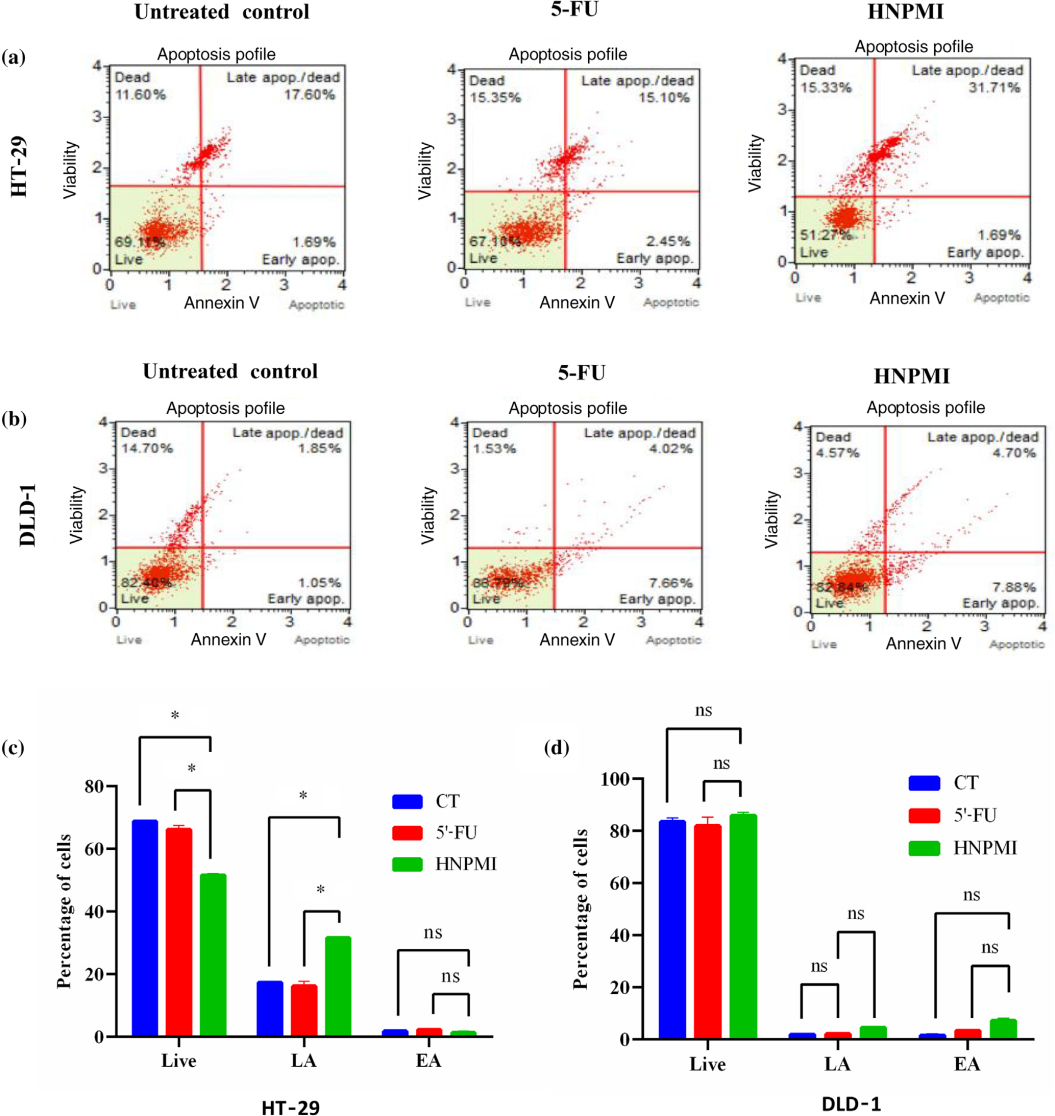
HNPMI induced apoptosis in HT‐29 and DLD‐1 colorectal cancer (CRC) cells. HT‐29 and DLD‐1 cells were treated with HNMPI (30 μM; 24 h), and the percentage of apoptosis was evaluated by Muse® Annexin V assay and Dead Cell Kit. Scatter plot representing the viability of cell population from HNPMI‐treated (a) HT‐29 cells and (b) DLD‐1 cells. Each quadrant was categorized as viable cells (lower left), early apoptotic cells (lower right), late apoptotic cells (upper right) and necrotic cells (upper left). The quantitative representation of panels (a) and (b) shows the percentage of cells in different stages of apoptosis following HNPMI treatment of (c) HT‐29 cells and (d) DLD‐1 cells, with 5‐fluorouracil (5′‐FU) as the positive control. Data shown are means ± SEM from n = 6 independent values. *P < 0.05, significantly different as indicated; ns, no significant difference between groups; two‐way ANOVA with multiple comparisons. CT, untreated control; EA, early apoptosis; LA, late apoptosis.

To examine the effects of HNPMI on apoptosis, we analysed the transition of apoptotic cells using Annexin V 7‐AAD assay, using 5′‐FU as a positive control. The scatter plot graph illustrates the viable cells (Annexin V [−] and 7‐AAD [−]), the early‐phase apoptotic cells (Annexin V [+] and 7‐AAD [−]), the late‐phase apoptotic or dead cells (Annexin V [+]), and the necrotic cells (Annexin V [−] and 7‐AAD [+]). As shown in Figure 4a,c, the late apoptotic cell population of HT‐29 cells treated with 5'‐FU and HNPMI in the upper right quadrant increased significantly. Paradoxically, the HNPMI‐treated DLD‐1 cells showed increased fractions of early apoptotic and late apoptotic cells, compared with the control and 5'‐FU treated cells (Figure 4b,d). Altogether, these data imply that HNPMI has the potential to induce apoptosis, which varied based on the tumour stage of the CRC cells.

### 3.6 HNPMI altered the expression of apoptosis‐related proteins

**FIGURE 5:**
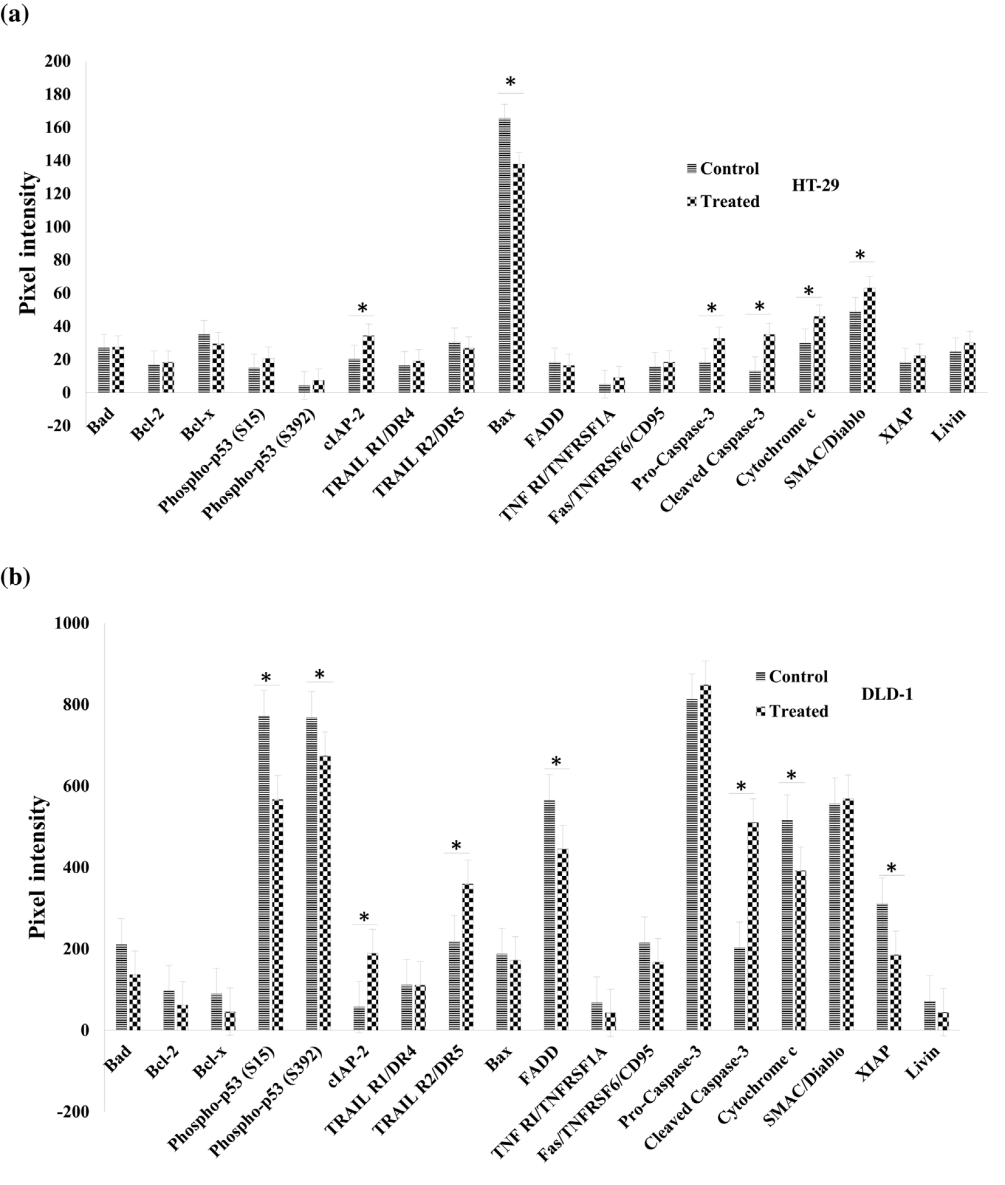
HNPMI modulated the expression of apoptosis‐related proteins in colorectal cancer (CRC) cells. Densitometric analysis using ImageJ showing the differentially expressed apoptotic related‐proteins in HNPMI treated HT‐29 cells and DLD‐1 cells. The bar diagrams (a) and (b) shows the pixel intensity in the expression of apoptosis‐related proteins in HNPMI‐treated HT‐29 cells (a) and (b) DLD‐1 cells, in comparison with the control cells (treated with 5'‐FU). The results are shown as arbitrary units of intensity and are expressed as means ± SEM from n = 6 independent experiments. *P < 0.05, significantly different as indicated; Student's *t* test.

From the previous analysis, HNPMI is reportedly associated with apoptosis, and to further evaluate this, a focussed antibody array was used to discern the modulation of apoptosis‐specific proteins. Densitometric analysis of differentially expressed apoptotic related‐proteins in HNPMI treated (Figure 5a) HT‐29 cells and (Figure 5b) DLD‐1 cells were carried out. The normalized relative expression of 35 apoptosis‐related proteins including effector and signalling molecules were evaluated in both cell lines.

HNPMI‐treated HT‐29 cells showed significant up‐regulation of 5 different apoptosis‐related proteins (Figure 5a) including pro‐caspase 3, cleaved caspase‐3, SMAC/Diablo, cytochrome C, and cIAP‐2, while Bax was significantly downregulated compared to 5'‐FU control. However, DLD‐1 cells displayed a varied protein profile with 3 up‐regulated proteins and 5 down‐regulated proteins, compared with 5'‐FU treatment (Figure 5b).  Phospho‐p53 (S15) and phospho p53(S392) was found to be upregulated in 5'‐FU treated DLD‐1cells than the HNPMI treated cells, whereas HT‐29 cells did not show any significant differences. These data suggest a possible cause of the resistance of DLD‐1 cells to 5'‐FU treatment (Chen et al., 2017; Inaba et al., 1998; Shieh et al., 1997; Van Der Jeught et al., 2018). While most of the apoptotic proteins showed no significant differences in DLD‐1 cells, Cleaved Caspase‐3 showed >1.6‐fold upregulation compared to the control (Figure 5b). Also, the expression of Pro‐Caspase‐3 was found to be significantly up‐regulated in HNPMI treated HT‐29 cells, whereas there was no significant difference in HNPMI treated DLD‐1 cells (Figure 5a, 5b). In contrast to HT‐29 cells, a <0.02‐fold decrease in phospho‐p53, and <1.5‐fold for cytochrome C was observed in DLD‐1 cells. BAX that promotes mitochondrial‐mediated cell death, were found to be downregulated in HNPMI treated HT‐29 cells, thus inducing caspase‐mediated apoptosis (Figure 5a).

### 3.7 HNPMI altered the transcriptional response of apoptosis‐specific genes

**FIGURE 6:**
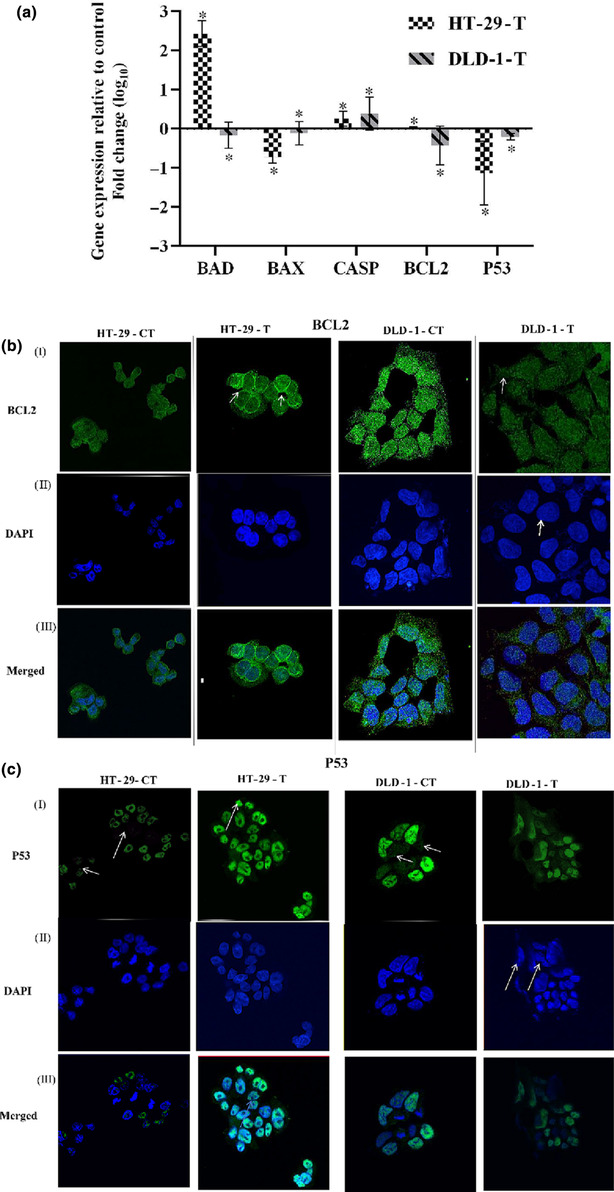
Effect of treatment with HNPMI on apoptosis‐related gene and intracellular BCL‐2 and p53 protein expression. (a) Quantitative expression of apoptosis‐related genes including BAD, BAX, CASP, BCL‐2 and p53, represented as fold change (log_*10*_) upon HNPMI treatment in HT‐29 and DLD‐1 cells. GAPDH was used as a loading control. Data shown are means ± SEM from n = 6 independent values. *P< 0.05, significantly different from control (zero log_10_ change); two‐way ANOVA). The fold changes were normalized against the control. The arrows in the confocal microscopy images (63×) showing the expression and localization of (b) BCL‐2 and (c) p53 protein expression using the corresponding primary antibodies in both HT‐29 and DLD‐1 cells.

In the present study, the mRNA level of the selected apoptotic‐related genes was evaluated by qPCR, to relate the changes in the protein expression obtained in the protein array. The proteins that showed significant changes in the apoptotic array such as the BCL‐2 family (BAD, BAX), p53 and CASP were selected for the analysis. The expressions of the target genes were compared with the housekeeping genes, GAPDH, in both cell lines, and the fold change was represented in Figure 6a. The level of pro‐apoptotic genes, BAD and CASP3, significantly increased in HT‐29 cells, whereas BAD decreased to a fold change of 0.2 and CASP3 increased 0.4‐fold in DLD‐1 cells. A 0.4‐fold decrease in BCL‐2 expression was detected in DLD‐1 cells, but with no significant change in HT‐29 cells. Also, there was a decreased fold change of about 0.75 and 0.1 for pro‐apoptotic gene BAX and a 1.1‐ and 0.3‐fold change for p53 expression for HT‐29 and DLD‐1 cells, respectively.

### 3.8 HNPMI influenced the intracellular localization of BCL‐2 and p53 protein expression

From the previous analysis, it appeared that the BCL‐2 family proteins and p53 protein were notably involved in the apoptosis process. Hence, further endogenous localization in intact HT‐29 and DLD‐1 cells was visualized using confocal microscopy to assess the effect of HNPMI. BCL‐2 protein was localized in the cytoplasm and around the nucleus in the untreated HT‐29 cells whereas, in the HNPMI‐treated cells, BCL‐2 protein while localized within the cytoplasm, this was predominantly around the nuclear periphery (Figure 6bI,II). In DLD‐2 cells, BCL‐2 localization was more dispersed around the nuclei in both control and treated cells. Based on the intensity of the fluorescence, the amount of BCL‐2 expression was qualitatively analysed. In both control and treated HT‐29 cells, BCL‐2 expression was perinuclear (+ in controls and ++ in HNPMI‐treated cells) with minimal localization in the cytoplasm (+ in control and ++ in HNPMI‐treated cells).

Of note, nuclear p53 expression was higher (+++++) in HNPMI‐treated HT‐29 cells than in the control cells (+++). Only a few of the control HT‐29 cells expressed nuclear p53 protein, which is indicated by the arrow in Figure 6cI. In HNPMI‐treated DLD‐1 cells, morphological changes were observed with reduced cell size, irregular cell shape, condensation of cytoplasmic content and the nucleus, all of which is an indication of apoptosis. Consistent with our previous data from the protein array and qPCR data, p53 expression was higher in untreated cells (+++++) than in the HNPMI‐treated DLD‐1 cells (Figure 6cII). Altogether, the data from these assays confirmed the role of anti‐apoptotic and pro‐apoptotic genes and proteins in inducing cell death and thus leading to apoptosis in CRC cells treated with the EGFR inhibitor, HNPMI.

### 3.9 HNPMI down‐regulated oncogenesis in CRC

**FIGURE 7:**
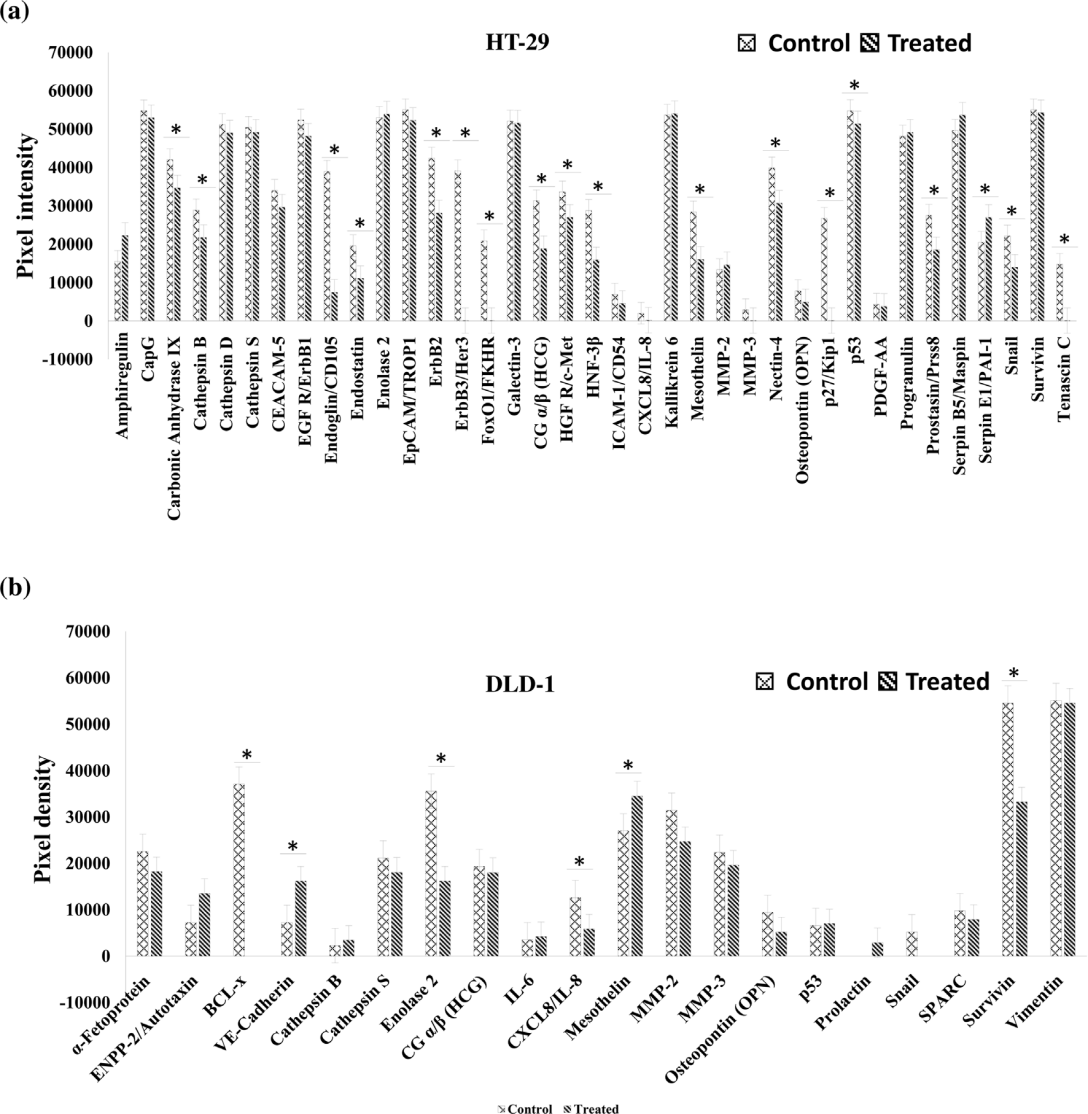
HNPMI regulated the expression of oncogene‐related proteins in colorectal cancer (CRC) cells. Densitometry analysis using ImageJ show the differentially expressed human XL oncogenic‐associated proteins in HNPMI‐treated (a) HT‐29 cells (a) and (b) DLD‐1 cells (b). The bar diagrams show the pixel intensities of significantly expressed oncogenic‐related protein in HNPMI‐treated HT‐29 cells (a) and DLD‐1 cells (b), in comparison with the control cells. Figures (a) and (b) include the differential expression of only detectable proteins out of 84 human cancer related proteins. The results are shown as arbitrary units of intensity and the data are expressed as means ± SEM from n = 6 independent experiments. *P < 0.05, significantly different from control; Student's *t* test.

Oncogenic transformation alters the protein expression of cells and their metabolism, which might be unique for a specific type of cancer. In order to validate the potential role of HNPMI in regulating the expression of oncogenic‐related proteins, the Proteome Profiler Human XL Oncology containing 84 human cancer‐related proteins was analysed. Notably, HNPMI strongly inhibited crucial anti‐apoptotic proteins including Snail, HGFR/c‐Met in HT29 cells and BCL‐X, EMT‐related protein, Survivin in DLD‐1 cells (Figure 7a, b). These data validate the effect of EGFR inhibitor, HNPMI in inhibiting anti‐apoptotic activity in the CRC cells.

### 3.10 Anti‐cancer evaluation of HNPMI in xenograft animal model

**FIGURE 8:**
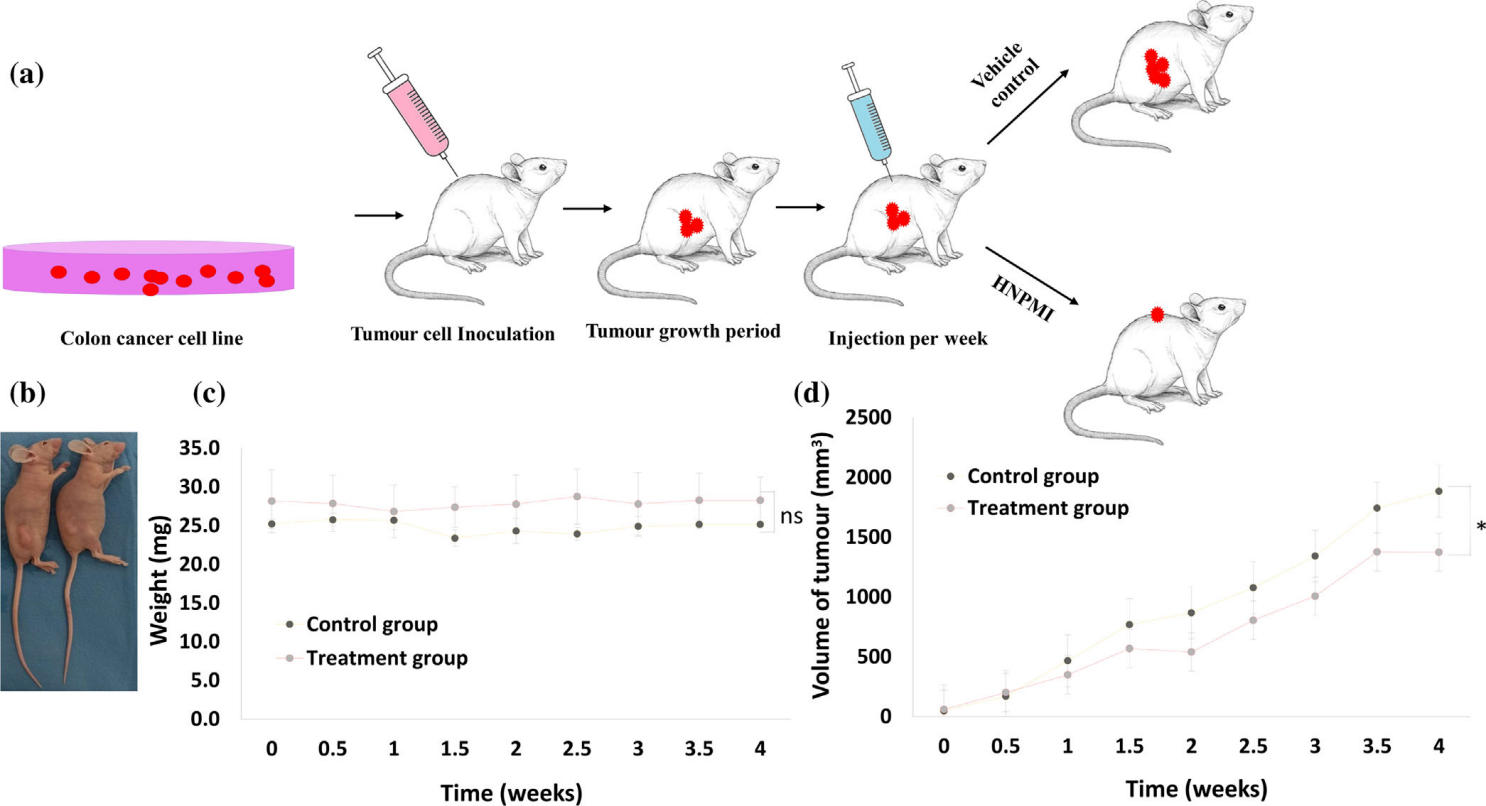
HNPMI inhibited the growth of colorectal cancer (CRC) cells in the xenograft mouse model. (a) Schematic representation of the development of xenograft mouse model injected intraperitoneally with CRC cells. (b) Non‐Scid xenograft animal model showing the development of tumour after 4 weeks after injection of colon cancer cells (left); HNPMI treatment reduced growth of tumour (right). (c) Body weight measurement in the HNPMI‐treated and control animal groups. (d) Measurement of tumour volume (mm^3^) using standard vernier calliper for a period of 4 weeks of treatment with HNPMI (120 mg·kg^−1^). Data shown are means ± SEM from n = 8 animals per group. Data were analysed by the χ^2^ test for the trend between groups. *P < 0.0001, significant difference between the control and treated groups; ns, no significant difference between the compared groups.

Anti‐colon cancer activity of HNPMI in vivo was evaluated in a xenograft mouse model. Figure 8a illustrates the process of development of xenograft mouse using HCT‐116 CRC cells with the experimental condition for a period of 28 days. The relative weight (mg) and relative tumour volume (mm^3^) were measured periodically (Figure 8b) to assess the effect of HNPMI on normal animals. The mean weight of the experimental animal was increased with a mean value of 0.03 mg from the first week of treatment, which was prolonged till the fourth week, and this might be due to the fluid accumulation and oedema (Figure 8c). Compared with the control group, the HNPMI‐treated (with the safety drug concentration) xenograft‐bearing mice showed a significantly increased trend of reducing the tumour volume (*P* < 0.0001) from the third injection till the ninth injection (Figure 8d).

## 4 DISCUSSION

The molecular diversity and chemo‐resistance of all cancers, especially CRC, demand an urgent need for novel therapeutics or candidate drug development. It is evident from the recent research, that the overexpression of EGFR and its influence on the downstream signalling mechanism in CRC plays a pivotal role in disease progression. Hence, the identification of a potential inhibitor for EGFR pathway is imperative for CRC therapy. Our previous investigation on alkylaminophenols such as THTMP, THMPP, and HNPMI as effective inhibitors of EGFR has been reported against breast cancer, glioblastoma, and osteosarcoma (Doan et al., 2016b; Nguyen et al., 2021; Palanivel et al., 2020), which motivated us to unravel its potential against CRC.

But a greater understanding of the molecular mechanism of action of these alkylaminophenols is necessary to uncover their ability to induce apoptosis mediated by potential EGFR inhibitors. *N*‐heterocycle‐derived compounds that selectively induce apoptosis are envisioned for the development of anti‐cancer drugs (Tandon et al., 2019). In the present investigation, the three alkylaminophenols of interest THTMP, THMPP, and HNPMI were investigated for their anti‐cytotoxicity effect against various types of cancer cell lines, including liver, colon, and prostate, revealing their ability to have a generic effect.

The dynamic morphological changes in the cell culture environment, including pyknosis, and karyorrhexis followed by crowding of apoptotic bodies and apoptotic shrinkage (Galluzzi et al., 2007), are the key cellular and molecular mechanism of apoptotic changes. Analogously, HNPMI exerted morphological changes causing blebbing and endonucleolytic cleavage of chromosomal DNA in both HT‐29 and DLD‐1 colon cancer cell lines. Several other natural and synthetic indole derivatives have been found to induce apoptosis or cell cycle arrest in various cancer cells, for example, HT‐29 and Caco‐2 colon cancer cells (Esmaeelian et al., 2013); and the HCT‐116 colon cancer cell line (Tischlerova et al., 2017). All these data ratify the potential of HNPMI in inducing apoptosis, which is commonly evaded by most cancer cells.

To check the ability of HNPMI in regulating the genes and proteins involved in apoptosis, gene expression profiling was performed in CRC cell lines. Notably, BAX, a cell death regulator that regulates the intrinsic pathway of apoptosis was inhibited by HNPMI in HT‐29 cell line. Similar data was also observed in BAX (‐) and p53 (‐) tumours showing greater response to 5'‐FU treatment (Hector & Prehn, 2009). Interestingly, HNPMI increased the expression of pro‐caspase 3 in HT‐29 cells and cleaved caspase 3 in both cell lines, an executioner endopeptidase (Porter & Jänicke, 1999), mediates proteolysis of crucial proteins involved in apoptosis. Thus, HNPMI being an effective regulator of the apoptosis signalling pathway, reduced the expression of the BCL‐2 gene in DLD‐1 cells (see Figure 6) and which in turn forms an apoptosome complex with BAX, and thus regulates the downstream initiator caspase‐3, thereby promoting apoptosis. Also, Smac/Diablo which promotes apoptosis by inhibiting IAP, an inhibitor of apoptosis (van Loo et al., 2002; Schimmer, 2004), was exclusively observed to be overexpressed in HNPMI treated HT‐29 cells. It was reported by (Alam et al., 2022) that potential EGFR inhibitors could regulate the BAX/BCL‐2 cascade in non‐small cell lung carcinoma (NSCLC). Similarly, in our previous in‐silico analysis (Palanivel et al., 2020), HNPMI was found to exhibit bimolecular interaction with EGFR, thereby inducing apoptosis through the EGFR mediated signaling pathway in breast cancer cells. Thus, it was resolved that HNPMI could potentially regulate the CRC disease progression, especially in HT‐29 cells, through the EGFR‐mediated BCL‐2/BAX mediated cascade, thus regulating apoptosis by the intrinsic apoptotic pathway.

The protein p53, a nuclear transcription factor with pro‐apoptotic function was also found here to be regulated by exposure of CRC cells to HNPMI. Of note, the expression of nuclear p53 protein was higher in HNPMI‐treated HT‐29 cells than in the control cells, which is the reverse of normal physiological conditions. This increased nuclear p53 might be due to the DNA damage causing post‐translational modifications converting the latent p53 to an active form within the cellular nucleus (Lacroix et al., 2006; Vogt Sionov & Haupt, 1999). In comparison, in HNPMI‐treated DLD‐1 cells, levels of nuclear p53 were decreased, which could possibly be caused by proteasomal degradation, mediated largely by RING‐finger type E3 ubiquitin‐protein ligase MDM2, and which further maintained p53 in a latent form (Honda et al., 1997; Kubbutat et al., 1997). It was reported that p53 was overexpressed in more than 70% of tumours in poorly differentiated colorectal carcinomas to less than 30% in those with well or moderately well differentiated tumours (Kapiteijn et al., 2001). There are also reports on the clinical and pathological differences between right and left side colorectal tumours. Thus, from our analysis, it was revealed that the differences in the expression of nuclear p53 in HNPMI‐treated HT‐29 and DLD‐1 cells might be due to the different staged CRC cell lines, which implies that the pathogenesis also might vary (Rosati et al., 2004). Moreover, several studies have reported that p53 and EGFR were overexpressed in CRC and HNPMI, acting as an effective inhibitor of EGFR, could conceivably induce or suppress the expression of p53 in different staged CRC cell lines, thereby inducing DNA damage and apoptosis.

To conclude, our results suggest that HNPMI acts as an effective EGFR inhibitor and exhibits potential anti‐cytotoxicity and anti‐proliferative activity in CRC cells by inducing the intrinsic apoptotic pathway and oncogenesis pathway. Thus, HNPMI could be used as a potential therapeutic agent for the treatment of early and late‐stage metastatic colon cancer. Besides its promising anti‐CRC property, the core structure of HNPMI may be further developed to improve EGFR inhibition and treat CRC‐resistant populations, particularly to overcome the resistance mechanism of CRC through the regulation of BCL‐2/BAX and p53 signalling pathways in colon cancer cells.

## AUTHOR CONTRIBUTIONS

**Jeyalakshmi Kandhavelu:** Investigation; In vitro methodology; validation (lead); writing—original draft (equal); writing—review and editing. **Kumar Subramanian:** Investigation; In vitro methodology; validation (equal); writing—original draft (equal); writing—review and editing. **Vivash Naidoo:** Investigation (equal); In vitro methodology; validation; writing—original draft (equal); writing—review and editing. **Giulia Sebastianelli:** Investigation; In vitro methodology; validation; writing—original draft (supporting); writing—review and editing. **Phuong Doan:** Investigation; In vitro methodology; validation (supporting); writing—original draft; writing—review and editing. **Saravanan Konda Mani:** computational analysis; writing—original draft; writing—review and editing. **Hande Yapislar:** Investigation (supporting); In vivo methodology, funding acquisition; project administration; resources; writing—review and editing. **Ebru Haciosmanoglu:** Investigation; In vivo methodology, funding acquisition; project administration; resources; writing—review and editing. **Leman Arslan:** Investigation; In vivo methodology, funding acquisition; project administration; resources; writing—review and editing. **Samed Ozer:** Investigation; In vivo methodology, funding acquisition; project administration; resources; writing—review and editing. **Ramesh Thiyagarajan:** validation; writing—original draft (supporting); writing—review and editing. **Nuno R. Candeias:** synthesized and characterized the compounds; funding acquisition (supporting); resources; supervision; writing—review and editing. **Clement Penny:** funding acquisition; project administration; resources; supervision; writing—review and editing. **Meenakshisundaram Kandhavelu:** Conceptualization (equal); data curation; formal analysis; funding acquisition (lead); project administration; supervision; validation; writing—original draft (lead); writing—review and editing (lead). **Akshaya Murugesan:** Conceptualization; data curation; formal analysis (lead); funding acquisition; project administration (lead); resources; supervision; writing—review and editing (lead).

## CONFLICT OF INTEREST STATEMENT

The authors declare no conflicts of interest.

## DECLARATION OF TRANSPARENCY AND SCIENTIFIC RIGOUR

This Declaration acknowledges that this paper adheres to the principles for transparent reporting and scientific rigour of preclinical research as stated in the *BJP* guidelines for Design and Analysis, Immunoblotting and Immunochemistry, and Animal Experimentation and as recommended by funding agencies, publishers and other organizations engaged with supporting research.

## Supporting information


**Table S1:** The half maximal inhibitory concentration (IC_50_) value of HNPMI, THTMP and THMPP treated PC‐3, Caco‐2, and HepG2 cancer cell lines.

## Data Availability

We declare that the data supporting the findings of this study are available within the paper and its supporting information and from the corresponding authors upon request.
