# Supplementary material for: A novel EGFR inhibitor, HNPMI, regulates apoptosis and oncogenesis by modulating BCL‐2/BAX and p53 in colon cancer
Source: Br J Pharmacol. 2023 Sep 12;181(1):107–24. doi: 10.1111/bph.16141 (PMC10952184; doi:10.1111/bph.16141)
Supplement: Supplementary file 1 — Table S1: The half maximal inhibitory concentration (IC50) value of HNPMI, THTMP and THMPP treated PC‐3, Caco‐2, and HepG2 cancer cell lines. [file BPH-181-107-s001.docx]

**Supplementary File**

**A novel EGFR Inhibitor, HNPMI regulates apoptosis and oncogenesis by modulating Bcl-2/Bax and p53 in colon cancer**

Jeyalakshmi Kandhavelu^1^, Kumar Subramanian^1^, Vivash Naidoo^1^, Giulia Sebastianelli^2^, Phuong Doan^2,3,4^, Saravanan Konda Mani^5^, Hande Yapislar^6^, Ebru Haciosmanoglu^7^, Leman Arslan^8^, Samed Ozer^6^, Ramesh Thiyagarajan^9^, Nuno R. Candeias^10,11^, Clement Penny*^1^, Meenakshisundaram Kandhavelu*^2,3,4^, Akshaya Murugesan^2,12*^

^1^Oncology Division, Faculty of Health Sciences, University of the Witwatersrand, Parktown 2050, Johannesburg, South Africa.

^2^Molecular Signalling Lab, Faculty of Medicine and Health Technology, BioMediTech, Tampere University and Tays Cancer Centre, P.O. Box 553, 33101 Tampere, Finland.

^3^BioMediTech Institute and Faculty of Medicine and Health Technology, Tampere University, Arvo Ylpön katu 34, 33520 Tampere, Finland.

^4^Science Center, Tampere University Hospital, Arvo Ylpön katu 34, 33520 Tampere, Finland.

^5^Research and Publication Wing, Bharath Institute of Higher Education and Research, Chennai – 600073, Tamil Nadu, India

^6^Acibadem University School of Medicine, Department of Physiology, 34684, Atasehir, Istanbul, Turkey

^7^BezmialemVakıf University, School of Medicine, Department of Biophysics, 34093 Fatih, Istanbul, Turkey

^8^BezmialemVakıf University, School of Medicine, Department of Physiology, 34093 Fatih, Istanbul, Turkey

^9^Department of Basic Medical Sciences, College of Medicine, Prince Sattam Bin Abdulaziz University, Al-Kharj, 11942, Kingdom of Saudi Arabia

^10^LAQV-REQUIMTE, Department of Chemistry, University of Aveiro, 3810-193, Aveiro, Portugal

^11^Faculty of Engineering and Natural Sciences, Tampere University, Korkeakoulunkatu 8, 33101 Tampere, Finland

^12^Department of Biotechnology, Lady Doak College, Thallakulam, Madurai – 625002, India.

Supplementary Table 1: The half maximal inhibitory concentration (IC_50_) value of HNPMI, THTMP and THMPP treated PC-3, Caco-2, and HepG2 cancer cell lines.

| Alkylaminophenols | Prostate | Colon | Liver |
| --- | --- | --- | --- |
|  | PC3 | Caco2 | HepG2 |
| HNPMI | 38.7 ± 1.1 | 28.1 ± 1.8 | 42.3 ± 8.9 |
| THTMP | 25.7 ± 6.1 | 57.6 ± 4.1 | 26.4 ± 2.3 |
| THMPP | > 100 | 38.5 ± 1.9 | 47.2 ± 1.4 |
